# Flavones: From Biosynthesis to Health Benefits

**DOI:** 10.3390/plants5020027

**Published:** 2016-06-21

**Authors:** Nan Jiang, Andrea I. Doseff, Erich Grotewold

**Affiliations:** 1 Center for Applied Plant Sciences, The Ohio State University, Columbus, OH 43210, USA; jiang.1359@osu.edu; 2 Department of Molecular Genetics, The Ohio State University, Columbus, OH 43210, USA; doseff.1@osu.edu; 3 Department of Physiology and Cell Biology, 305B Heart and Lung Research Institute, The Ohio State University, Columbus, OH 43210, USA

**Keywords:** flavone, flavone synthase, biological activities, health benefits, cytochrome P-450, Fe^2+^/2-oxoglutarate-dependent dioxygenases, *C*-glycosyl transferases

## Abstract

Flavones correspond to a flavonoid subgroup that is widely distributed in the plants, and which can be synthesized by different pathways, depending on whether they contain *C*- or *O*-glycosylation and hydroxylated B-ring. Flavones are emerging as very important specialized metabolites involved in plant signaling and defense, as well as key ingredients of the human diet, with significant health benefits. Here, we appraise flavone formation in plants, emphasizing the emerging theme that biosynthesis pathway determines flavone chemistry. Additionally, we briefly review the biological activities of flavones, both from the perspective of the functions that they play in biotic and abiotic plant interactions, as well as their roles as nutraceutical components of the human and animal diet.

## 1. Introduction

**Figure 1:**
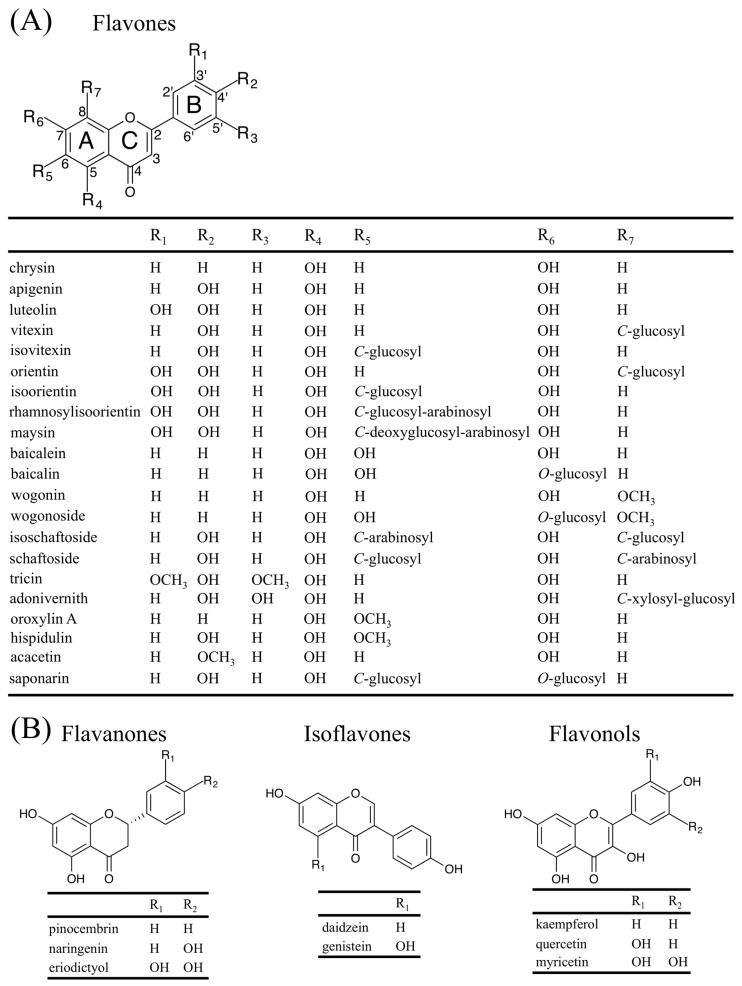
Structures of some important flavonoids discussed in this review: (**A**) flavones, including aglycones, *C*-/*O*-glycosyl flavones, and *O*-methylated flavones; and (**B**) selected flavonoids mentioned in the text belonging to the flavanone, isoflavone, and flavonol groups.

Flavonoids represent a large subgroup of the phenolic class of plant specialized metabolites. They are widely distributed throughout the plant kingdom [1]. The basic flavan skeleton that forms all flavonoids is a 15-carbon phenylpropanoid core (C_6_-C_3_-C_6_ system), which is arranged into two aromatic rings (A and B) linked by a heterocyclic pyran ring (C) (Figure 1A). According to the oxidation status and saturation of the heterocyclic ring, flavonoids are divided into several groups, which include flavones, flavanones, isoflavones, flavonols, 3-deoxy flavonoids, and anthocyanins [1]. Flavones comprise one of the largest groups, which are characterized by the presence of a double bond between C-2 and C-3, and the attachment of the B ring to C-2 (Figure 1A; [1]). As is the case with other flavonoids, flavones have a diversity of functions that have contributed in making plants adapt to a terrestrial environment including: (i) protection against UV radiation [2] and oxidative stress [3]; (ii) interspecies interactions (pathogen resistance [4], symbiosis [5], protection from herbivory [6], and allelopathy [7]); and (iii) plant development (copigmentation with anthocyanins [8] and lignification [9]). In addition to their physiological, biochemical, and ecological functions to plants, flavones also exert biological activities on animals, providing important nutritional value [10]. Key to fulfilling these multiple roles, flavones are characterized by a high degree of chemical diversity provided by modifications of the chemical backbone, which include hydroxylation, *O*-/*C*-glycosylation, *O*-methylation, and acylation [1]. This review focuses on the multiple and specialized metabolic pathways responsible for flavone biosynthesis, discusses their roles in plant biology and briefly summarizes the recent progress towards establishing the mechanisms by which flavones provide health benefits.

## 2. Flavone Biosynthesis: Multiple and Specialized Biosynthesis Pathways Result in Flavone Chemical Diversity

### 2.1. Multiple Enzymes Can Form the Flavone Backbone

#### 2.1.1. Biosynthesis of Flavone Precursors

**Figure 2:**
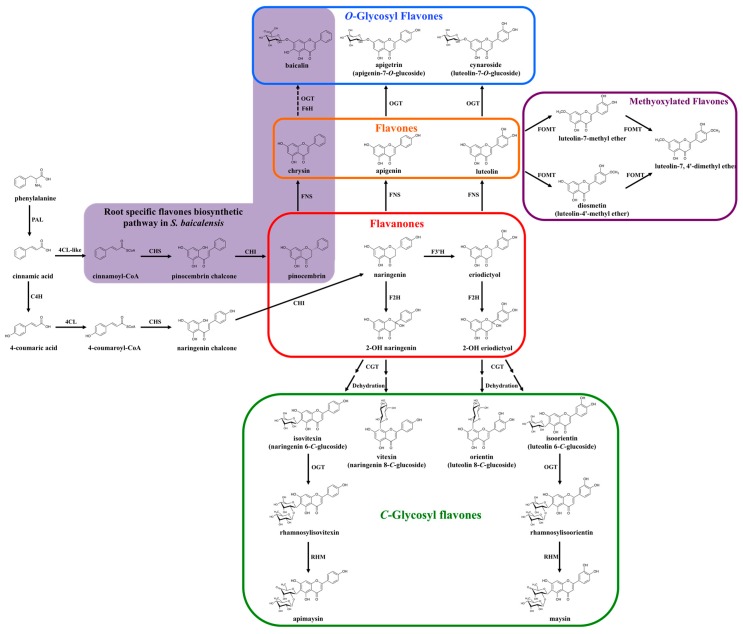
Multiple and specialized flavone biosynthetic pathways. Purple path: Newly identified biosynthetic pathway for root-specific flavones in *Scutellaria baicalensis*. PAL: phenylalanine ammonia lyase; C4H: cinnamate-4-hydroxylase; 4CL: p-coumaroyl-CoA ligase; 4CL-like: cinnamic acid specific CoA ligase; CHS: chalcone synthase; CHI: chalcone isomerase; FNS: flavone synthase; F2H: flavanone-2-hydroxylase; F3′H: flavanone-3′-hydroxylase; F6H: flavanone-6-hydroxylase; OGT: *O*-glycosyltransferase; FOMT: flavonoid *O*-methyltransferase; CGT: *C*-glycosyltransferase; RHM: UDP-rhamnose synthase.

In most species, the biosynthesis of the flavone backbone originates from the general phenylpropanoid pathway followed by the flavonoid biosynthetic branch [11]. As part of the phenylpropanoid pathway, phenylalanine is deaminated to cinnamic acid by phenylalanine ammonia-lyase (PAL). p-coumaric acid is then formed from cinnamic acid by cinnamic acid 4-hydroxylase (C4H), which catalyzes the introduction of an hydroxyl group on the phenyl ring. The carboxyl group of p-coumaric acid is then activated to form p-coumaroyl-coenzyme A (CoA), through the formation of a thioester bond with CoA, a process catalyzed by p-coumaroyl-CoA ligase (4CL) (Figure 2, [11]).

After this, p-coumaroyl-CoA is condensed with three malonyl-CoA to form chalcone by the chalcone synthase (CHS) enzyme (Figure 2, [12]). Chalcone synthases belong to the type III polyketide synthase family, present in most plant species [13]. The mechanism of CHS action demonstrated that as a starter molecule, p-coumaroyl-CoA binds to a cysteine residue in the active site of CHS and a tetraketide intermediate is formed subsequently by a series of decarboxylative condensation reactions that involves adding three malonyl-CoA extender molecules. Chalcone (4,2′,4′,6′-tetrahydroxychalcone) is then produced by the intramolecular cyclization of the tetraketide intermediate [13].

Chalcone is subsequently isomerized into a flavanone (e.g., naringenin or eriodictyol) in a stereo-specific fashion by the action of chalcone isomerase (CHI, Figure 2; [14]). CHI is a very efficient enzyme and highly specific to the chalcone substrate. However, recent studies have shown that the CHI fold is derived from fatty-acid-binding proteins, which have no enzymatic activity and associate with saturated fatty acids [15]. How this enzyme was co-opted to the flavonoid pathway remains unclear, although CHI enzymes (or their products) might participate in ways that are not well understood in intracellular transport processes [16].

Finally, flavones are synthesized from flavanones by the introduction of a double bond between the C-2 and C-3 positions by a group of enzymes known as flavone synthase (FNS; Figure 2; [17]). Flavone synthases have the unique characteristic that two distinct enzyme types can catalyze the conversion of equivalent substrates (flavanones) to identical products (flavones), by rather different mechanism [17].

#### 2.1.2. Flavone Synthase I (FNSI) Class

The FNSI class belongs to the superfamily of soluble Fe^2+^/2-oxoglutarate-dependent dioxygenases (2-ODDs) and catalyzes the conversion of flavanones to flavones (Figure 2; [17]). The desaturation of the flavanones’ C-ring involves two steps consisting of the initial elimination of the C-3 β-configured hydrogen, followed by the elimination of the C-2 hydrogen [18]. The first FNSI enzyme was identified from leaflets of *Petroselinum crispum cv*. Activity assays confirmed that FNSI converted ^14^C-radiolabeled flavanones to the corresponding flavones without forming a detectable reaction intermediate [19]. Following these initial studies, FNSI enzymes were identified from a number of species of the *Apiaceae* family, and it was believed for many years that FNSI-type enzymes were restricted to the *Apiaceae* [20,21]. However, recent studies showed that the rice FNSI enzyme OsFNSI-1 converts the flavanone (*2S*)-naringenin into apigenin *in vitro* [22], indicating that 2-ODDs with FNS activity are more widely distributed than initially believed*.* Indeed, a FNSI enzyme (PaFNSI) was cloned from the liverwort *Plagiochasma appendiculatum* [23]. Recombinant PaFNSI not only had FNSI activity, but was also able to catalyze the conversion of flavanones to 2-hydroxyflavanones, and therefore displayed flavanone-2-hydroxylase (F2H) activity *in vitro* [23]. Further analyses indicated that Tyr240 was the critical residue for F2H activity, since the Tyr240Pro mutant converted naringenin to apigenin, but could not produce 2-hydroxynaringenin [23]. As described later in this review, these findings are significant because separate cytochrome P450 (CYP) enzymes can also have F2H or FNS (FNSII) activity.

Recently, FNSI enzymes have been characterized from maize (*Zea mays*) and Arabidopsis (*Arabidopsis thaliana*) [24]. ZmFNSI-1 and its Arabidopsis counterpart, AtDMR6, harbor *in vitro* FNSI activity. While *dmr6* Arabidopsis mutants show increased resistance to various pathogens including *Pseudomonas syringae*, *dmr6* plants transgenic for ZmFNSI-1 are equally susceptible to the pathogen as wild-type plants. The *dmr6* mutants accumulate higher salicylic acid levels than wild-type plants, and the increased salicylic acid levels present in *dmr6* are likely responsible for the increased tolerance to *Pseudomonas syringae* [25]. Thus, a feedback relationship between flavones (e.g., apigenin) and the salicylic acid metabolic pathways was proposed [24]. Possibly, to increase success in the plant, *P. syringae* (and other pathogens [26]) induce flavone accumulation, which results in the decrease of salicylic acid, and therefore increased pathogen susceptibility [27].

FNSI might have evolved from flavanone 3β-hydroxylases (FHTs), 2-ODDs that catalyze the conversion of (*2S*)-flavanones to (*2R*,*3R*)-dihydroflavonols [18], by gene duplication and functional diversification [20]. Gebhardt *et al.* (2007) demonstrated that site-directed mutagenesis of critical amino acids converted the activity of FHTs to that of FNSI. After homology modeling analyses between FHT and FNSI, seven critical amino acids were identified around the active site. Results of site-directed mutagenesis from one to seven substitutions of FHT indicated that a minimal of three amino acids (Ile131Phe, Met106Thr, and Asp195Glu or Ile131Phe, Leu215Val, and Lys216Arg) were sufficient to result in partial FNSI activity. All seven amino acid substitutions were sufficient to change the FHT activity completely to FNSI [20].

#### 2.1.3. Flavone Synthase II (FNSII) Class

FNSII enzymes correspond to oxygen- and NADPH-dependent cytochrome P450 (CYPs) membrane-bound monooxygenases, which are widespread among the higher plants [17]. So far, all characterized FNSII enzymes belong to the CYP93B subfamily for dicots and to the CYP93G subfamily for monocots. Most FNSII enzymes convert flavanones into flavones directly by introducing a double bond between the C-2 and C-3 residues in flavanones, as we described earlier for FNSI [17]. FNSII enzymes were first identified from snapdragon and Torenia petal cDNA libraries. AFNS2 (CYP93B3) and TFNS5 (CYP93B4) catalyzed the direct conversion of flavanones to flavones [28]. Later, an oxylipin-induced FNSII (CYP93B16) from soybean (*Glycine max*) cell cultures was characterized by enzymatic assays [29]. For the CYP93G subfamily, rice CYP93G1 was the first characterized monocot FNSII [30]. Recently, three FNSII (LjFNSII-1.1, LjFNSII-2.1, and LmFNSII-1.1) from *Lonicera japonica* and *Lonicera macranthoides* were confirmed to convert eriodictyol and naringenin directly to luteolin and apigenin, respectively. Site-directed mutagenesis analyses showed that the basic amino acid Lys242 is important for FNSII catalytic activity [31].

#### 2.1.4. 2-Hydroxyflavanones as Flavone Precursors

Most flavones are synthesized by the direct conversion of flavanones, as described in the previous section. However, some flavones, particularly those harboring *C*-glycosylation, use instead 2-hydroxyflavanones as precursors, which are produced from flavanones by F2H CYP enzymes. For example, members from the *Fabaceae* were confirmed to have F2H activity (CYP93B1 from *Glycyrrhiza echinata* [32] and CYP93B10/11 from *Medicago truncatula* [33]). The two *M. truncatula* *FNSII* genes have distinct tissue-specific expression patterns, with *MtFNSII-1* (CYPB10) highly expressed in roots and seeds, while *MtFNSII-2* (CYPB11) is highly expressed in flowers and siliques [33]. In the *Poaceae*, a sorghum (*Sorghum bicolor*) pathogen-induced FNSII (CYP93G3) [34], CYP92G2 from rice [35], and CYP93G5 from maize [36], all have been shown to have F2H activity. Once flavanones are 2-hydroxylated by F2H enzymes, they appear to serve as substrates for *C*-glycosyl transferase enzymes, followed by a dehydration reaction (see Section 2.2). Table 1 lists all of the so far characterized FNSI, FNSII, and F2H plant enzymes.

#### 2.1.5. Evolutionary Relationships between Flavone Synthase II (FNSII), Flavanone-2-Hydroxylase (F2H), and Isoflavone Synthase (IFS) in the CYP93 Subfamily

Isoflavonoids are characteristic of the leguminous plants (*Fabaceae*) [41], but are also found in multiple other plant species [42]. Formation of the isoflavone skeleton requires two steps: The first one is a CYP-dependent oxidative aryl migration of flavanones to yield a 2-hydroxyisoflavanone. IFS facilitates the removal of the 3β-hydrogen from the flavanone substrate, followed by migration of the side phenyl (B ring) from C-2 to C-3 and final hydroxylation at C-2 to form 2-hydroxyisoflavanone [43]. The next one is the introduction of a double bond between C-2 and C-3, and is catalyzed by a dehydratase [44]. The first IFS (CYP93C2) was identified from Licorice [45]. Mutagenesis studies revealed that Ser310 and Leu371 regulate the aryl migration and the formation of the by-product 3-hydroxyflavanone [43,46]. In addition, Lys375 in CYP93C2 is essential for the aryl migration, since mutagenesis of Lys375 to Thr disrupted the aryl migration and generated only the by-product 3-hydroxyflavanone [41,46].

**Figure 3:**
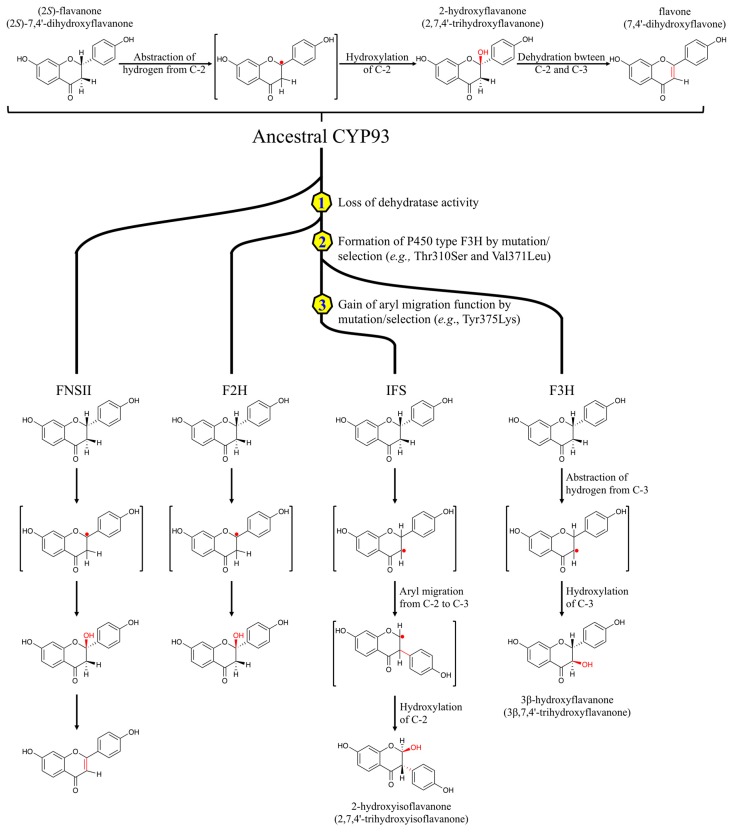
Proposed molecular evolutionary process of FNSII, F2H, IFS, and F3H from an ancestral CYP93 [43,46]. Amino acid numbers refer to positions in CYP93C2. FNSII: flavone synthase II; F2H: flavanone 2-hydroxylase; IFS: 2-hydroxyisoflavanone synthase; F3H: flavanone 3β-hydroxylase.

The ancestral CYP93 protein may have had FNS activity, converting flavanones to flavones directly (Figure 3) [46]. F2H might have evolved by a deletion of eight amino acid residues from the ancestral CYP93 protein, resulting in the lack of dehydratase activity, which introduces the double bond between C-2 and C-3 of the 2-hydroxylated flavanones [32]. During the radiation of the Leguminosae, it was proposed that gene duplication was followed by mutations of Ser310 and Leu371 in one of the two paralogs. These mutations lead to flavanone 3β-hydroxylase activity, which removes a proton at C-3 of the substrate, instead of at C-2 [43]. Finally, the unique aryl migration function of IFS was generated by further mutations, specifically the replacement of Val with Lys at position 375 (Figure 3) [46].

### 2.2. Biosynthesis of O- and C-glycosyl Flavones

#### 2.2.1. Backbone or Decoration First?

In plants, most flavones exist decorated by methylation, glycosylation or other modifications. Glycosylation is one of the most common modifications and is crucial for multiple chemical properties characteristic of flavonoids in the cell, such as increased solubility and stability [1]. *O*-linked glycosides are the most common types of of glycosylations, in which the flavone backbone (aglycone) is linked to sugar moieties through one of the multiple -OH groups present in flavones (Figure 2) [47]. However, glycosylation can occur by direct linkage of carbon residues in the flavonoid and sugar backbones, resulting in *C*-glycosyl flavones [48]. Actually, *C*-glycosyl flavones and their derivatives have been found in almost all plant phyla, ranging from liverworts (*Frullania polysticta* [49] and *Plagiochila jamesonii* [50]), ferns (*Asplenium viviparum* [51] and *Trichomanes* [52]), to flowing plants including monocots (hair grass *Deschampsia antarctica* [53], barley *Hordeum vulgare* [54,55], and maize [56,57,58]) and dicots (black calla *Arum palaestinum* [59], weed silene *Silene conoidea* [60], yarrow *Achillea setacea* [61], wild hop *Bryonia alba* [62], colocynth *Citrullus colocynthis* [63], cucumber *Cucumis sativus* [64], bottle gentian *Gentiana andrewsii* [65], geraniums *Pelargonium* [66], *Scutellaria baicalensis* [67], jequirity *Abrus precatorius* [68], *Glycyrrhiza eurycarpa* [69], lupine *Lupinus hartwegii* [70], shy plant *Mimosa pudica* [71], orchid *Ornithocephalus* [72], purple passionflower *Passiflora incarnate* [73], milkworts *Polygala telephioides* [74], globeflower *Trollius ledebourii* [75], quince *Cydonia oblonga* [76], lemon *Citrus* [77], and field pansy *Viola arvensis* [78]).

Compared with *O*-linked glycosides, *C*-glycosides are much more stable, since C-C bonds are largely resistant to glycosidase action or acid hydrolysis [79]. Intramolecular C-C phenol coupling reactions were found in the biosynthesis of isoquinoline alkaloid (CYP80G2, [80]) and morphine (CYP719B1, [81]). Some members from the lyase family (e.g., norcoclaurine synthase [82], strictosidine synthase [83], and tyrosine phenol lyase [84]) and oxidoreductases [85,86,87] also can catalyze the formation of C-C bonds.

**Figure 4:**
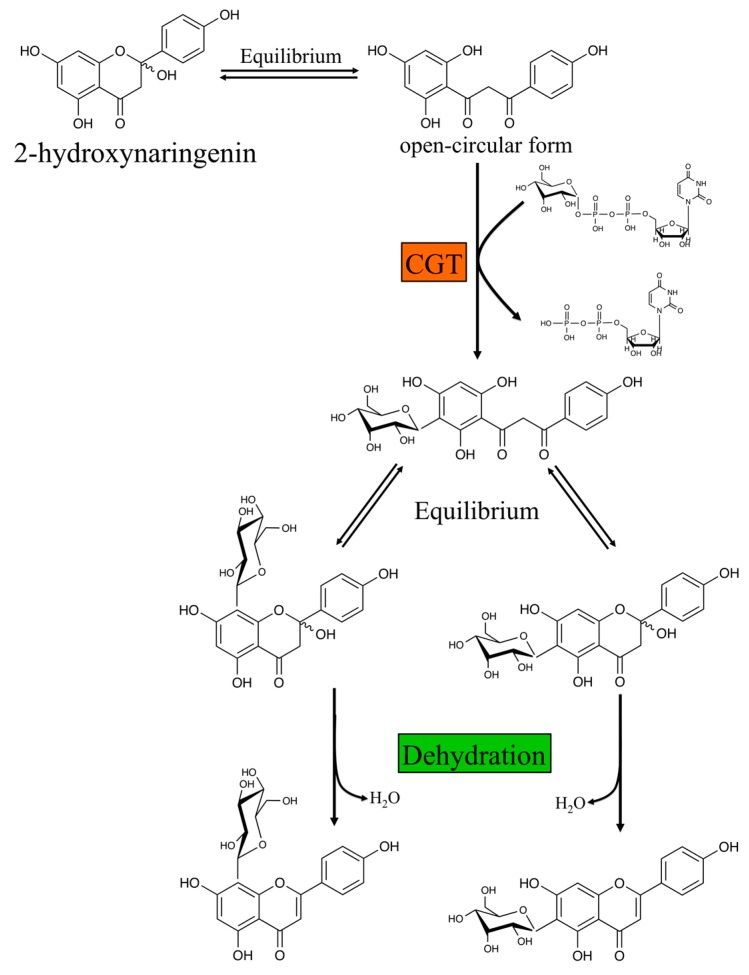
A proposed mechanism for the conversion of 2-hydroxynaringenin to vitexin and isovitexin by CGT and dehydration [79]. CGT: *C*-glycosyltransferase.

Two independent metabolic pathways are responsible for the formation of *O*- and *C*-glycosyl flavones [35,36]. *O*-glycosylation occurs after the flavone backbone is generated by FNSI or FNSII, depending of the plant species. This is called the “backbone first” pathway. In contrast, *C*-glycosyl flavone biosynthesis requires two stpes. First, the flavanone substrates are hydroxylated by F2H to form 2-hydroxylflavanones. The open-circular (dibenzoylmethane) form of the 2-hydroxylflavanone is then glycosylated by a glycosyltransferase. After conjugation of the sugar moiety, the closed-circular form of the products (2-hydroxylflavanone *C*-glycosides) is dehydrated to produce the corresponding flavone *C*-glycosides, in a process referred as “decoration first” pathway (Figure 4) [79].

#### 2.2.2. *O*- and *C*-glycosyl Transferases

*O*- and *C*-glycosylation are catalyzed by UDP-glycosyltransferases (UGTs), members of the glycosyltransferase family 1 (GT1), and use nucleotide sugars as donors [88]. Genes encoding for enzymes that catalyze flavone *C*-glycosylation at either C-6 or C-8 have been identified from rice (OsCGT) [79], maize (UGT708A6) [89], *Fagopyrum esculentum M.* (buckwheat UGT708C1 and UGT708C2) [90], soybean (UGT708D1) [91], *Gentiana triflora* (Japanese gentian GtUF6CGT1) [92], and *Mangifera indica* (Mango MiUGT13) [93]. Residues His20, Asp85, and Arg292) within the N-terminal acceptor-binding pocket seem necessary for the *C*-glycosyl transferase (CGT) activity. Substitution of Asp85 and Arg292 with Ala in UGT708D1 resulted in loss of CGT activity, while the His20Ala mutant protein also lost CGT activity but aquired a new *O*-glycosyl transferase (OGT) activity. Thus, Asp85 and Arg292 are essential for enzymatic activity, while Ala substitution of His20 also resulted in loss of CGT activity but aquired a new *O*-glycosyl transferase (OGT) activity [91]. Maize UGT708A6 is a bifunctional *O*-/*C*-glycosyltransferase that catalyzes the formation of both *C*- and *O*-glycoside links with 2-hydroxyflavanones and flavanones, respectively [89]. Protein sequence alignment among maize UGT708A6 with known UGTs showed that UGT708A6 not only contains the conserved residues (Asp91 and Arg287), which are restricted to the 2-hydroxyflavanone CGTs, but also has the conserved His-Asp residues corresponding to the active site of OGTs [89,91]. Interestingly, Japanese gentian GtUF6CGT1 can catalyze flavone *C*-glucosylation by directly adding a glucose group to the C6 position of the flavone skeleton, without the 2-hydroxyflavanone intermediate [92]. Recently, the novel benzophenone *C*-glycosyltransferase (MiCGT) from mango, which is involved in the biosynthesis of mangiferin, showed unusual substrates (both sugar acceptor and donors) and catalytic promiscuity [93]. MiCGT formed only *C*-glycosides with 2,4,6-tri-hydroxy acceptors, both *C*- and *O*-glycosides with 2,4-di-hydroxyl acceptors, and only *O*-glycosides with 2- or 4-mono-hydroxy acceptors. Thus, results from this study indicate that the number and position of the electron-donating hydroxy groups in the A-ring provide critical determinants for the *C*- or *O*-glycosylation capacity of MiCGT [93].

The biosynthesis of the maize *C*-glycosylflavone maysin provides a good example of the sophistication of the pathways involved in flavone formation (Figure 2). Maysin is a maize host-plant resistance factor against the corn earworm (*Helicoverpa zea*) and fall armyworm (*Spodoptera frugiperda*) [94,95,96,97]. Maysin derives from the conversion of eriodictyol to 2-hydroxy eriodictyol by a flavanone 2-hydroxylase (F2H1, CYP93G5) [36]. Subsequently, 2-hydroxy eriodictyol is *C*-glycosylated by UGT708A6 [89], and dehydrated to form isoorientin (luteolin 6-*C*-glucoside). It is unclear yet if this dehydration is spontaneous or enzymatic. Two additional enzymatic steps are required for the conversion of isoorientin to maysin. A rhamnose residue is first incorporated onto the glucose moiety of isoorientin to form rhamnosylisoorientin (isoorientin 2″-*O*-rhamnoside) by a rhamnosyl transferase. Maysin is then formed by dehydration of the glucose moiety in rhamnosylisoorientin to 4-keto-6-deoxy glucose by a rhamnose synthase [98]. The identification of the last two steps of the pathway was possible due to the availability of mutants which accumulate 3-deoxyanthocyanins, providing a salmon color to the silks, and mutants in two salmon silk loci had been previously identified [99,100].

In some species, mono-glycosylated flavones can be further decorated to form di-*C*-glycosyl flavones. A number of di-*C*-glycosyl flavones possessing *C*-glucosyl and *C*-arabinosyl moieties were identified as allelopathic compounds in *Desmodium incanum* against the parasitic weed *Striga hermonthica* [101]. Sequential *C*-glycosylation steps are involved in the biosynthesis of these di-*C*-glycosyl flavones. First, the mono-glucosylated 2-hydroxyflavanone intermediates were formed from *C*-glucosylation of 2-hydroxynaringenin with further *C*-arabinosylation. Subsequently, the 2-hydroxyflavanone products were dehydrated to yield di-*C*-glycosyl flavones, including two 6,8-di-*C*-glycosylated isomers, isoschaftoside and schaftoside [102].

#### 2.2.3. Evidence for Dehydratase Activity

The last step in the formation of *C*-glycosyl flavones is the dehydration of the 2-hydroxyflavanone, so far poorly understood. Both enzyme-catalyzed reactions by dehydratases and spontaneous dehydration were observed, and may co-exist. Dehydration of the maize glucosylated product is likely to be spontaneous, since no 2-hydroxyflavanone *C*-glycosides were identified following the in vitro reaction [89]. On the other hand, specific dehydratases seem to operate in buckwheat and rice [79,90]. If the dehydration process were spontaneous, all glycosylated 2-hydroxylflavanones (equal molar equivalents of the 6*C*- and 8*C*-glucosyl 2-hydroxylflavanones) should be dehydrated to the corresponding flavones. In contrast, flavone 6*C*-glucosides preferentially accumulated *in planta* [90]. These results suggest that a dehydratase activity is required to regulate the regioisomer ratios, which resulted in flavone 6*C*-glucosides to accumulate in buckwheat and rice [79,90]. A similar situation is found in the dehydration of 2-hydroxyisoflavanones to form isoflavones by 2-hydroxyisoflavanone dehydratase. Two 2-hydroxyisoflavanone dehydratases were characterized from *Glycyrrhiza echinata* and soybean [103]. Since the spontaneous dehydration of 2-hydroxyisoflavanone was slow and negligible compared to the enzyme-catalyzed reaction, the dehydration of 2-hydroxyisoflavanones to form isoflavones was proposed to be primarily enzyme dependent in plant cells [103].

### 2.3. Dedicated Biosynthetic Pathway for 4′ Deoxyflavones (B-Ring Deoxyflavonoids)

B-ring deoxyflavonoids that lack the -OH group at the 4′ position of the B-ring, were first discovered by chemical analysis of chitin-elicited cactus *Cephalocereus senilis* cultures [104]. Pinocembrin, a non-hydroxylated flavanone, is converted from cinnamic acid through cinnamoyl-CoA and 2′,4′,6′-trihydroxychalcone intermediates as a consequence of the characterization of a single form of CoA ligase being active on both cinnamate and 4-coumarate. Unusually, CHS and CHI from *C. senilis* cultures were active with cinnamoyl-CoA and 2′,4′,6′-trihydroxychalcone, respectively [104]. To synthesize these unusual deoxyflavonoids, metabolic compartmentalization between PAL and CoA ligase has been proposed, bypassing the C4H reaction and leading to the formation of cinnamoyl-CoA, which was incorporated into B-ring deoxyflavonoids via non-discriminating CHS and CHI activities [104].

Recently, this specialized pathway for 4′ deoxyflavonoids biosynthesis was characterized from roots of *Scutellaria baicalensis* [40], a traditional Chinese medicinal plant, whose active compounds include baicalin, baicalein, wogonoside, wogonin, neobaicalein, visidulin I and oroxylin A. Pinocembrin, rather than naringenin (mono-hydroxylated flavanone), was identified as the intermediate in the formation of these root-specific 4′-deoxyflavones. Several key enzymes have been characterized in this specialized pathway, including a cinnamic acid specific CoA ligase (4CL-like), which uses cinnamic acid to form cinnamoyl-CoA (bypassing 4-coumaric acid for addition of CoA), a specific isoform of CHS in roots, and a FNSII which is specific for pinocembrin and which generates chrysin as a product. This root-specific flavones pathway was successfully reconstituted in tobacco by co-expression of the characterized enzymes [40]. These findings revealed a great level of plasticity in the pathways involved in generation of flavonoids with rare modifications.

### 2.4. Other Flavone Modifications

Plants also produce other specialized flavones derivatives by hydroxylation, *O*-methylation/*O*-demethylation, and acylation [1]. Some hydroxylated flavones are methylated by *O*-methyltransferases (OMTs), which transfer methyl groups from *S*-adenosyl-l-methionine (donor) to the -OH groups of flavones (acceptors). In the past few years, the biosynthesis of poly-methoxylated flavones was defined in sweet basil (*Ocimum basilicum*) [105], an aromatic herb belonging to the mint family. The glandular trichomes accumulating lipophilic flavones with hydroxyl groups at positions 5, 6, 7, 8, and 4′ and up to four *O*-methyl residues at positions 6, 7, 8 and 4′ in this plant provided an ideal system to investigate the biosynthesis of poly-methoxylated flavones. First, a set of cation-independent methyltransferases (ObFOMT1-6) which catalyze regioselective 6-, 7-, and 4′-*O*-methylations was identified [106]. Two flavone 8-*O*-methyltransferases (ObPFOMT-1 and ObF8OMT-1) catalyze *O*-methylation at C8 [107]. Compared to *O*-methylation, *O*-demethylation is very rare in plant specialized metabolism. A flavone 7-*O*-demethylase (ObF7ODM1), which regiospecifically catalyzes the 7-*O*-demethylation of methoxylated flavones (gardenin B and 8-hydroxysalvigenin) was identified [108]. Recently, certain sequential catalytic reactions were found in the biosynthesis of some polymethoxylated flavones (nevadensin and pilosin) in sweet basil. To synthesize nevadensin and pilosin, 7-*O*-methylated flavone was required to be formed first as a precursor for the following 6-hydroxylation by flavone-6-hydroxylase (CYP82D33). Subsequently, the 7-unmethylated flavones (nevadensin and pilosin) are generated by ObF7ODM1 through the removal of the 7-methyl group [109]. Some rice OMTs (e.g., ROMT9, ROMT15, and ROMT17) were also identified to be functional in tricin (3’,5’-dimethoxyflavone) biosynthesis [110,111].

## 3. Biological Activities of Flavones

### 3.1. Biological Activities of Flavones in Plants

#### 3.1.1. Abiotic Protection

Flavonoids, including flavones, provide a shielding mechanism against the harmful effects of UV-B radiation (280–315 nm) by accumulating in both the epidermises and the outermost cell layers of the mesophyll tissues. UV-B radiation causes cellular damage by generating photoproducts in DNA and direct damage to proteins and lipids [2]. Flavones have two major absorption peaks: in the 290–400 nm range provided by the cinnamoyl part of the molecule; and in the range 240–285 nm provided by the benzyol part [112]. Thus, plants take full advantage of these broad absorption spectra from the structure properties of flavones. For example, in developing leaves of barley, saponarin (isovitexin-7-*O*-glucoside) and lutonarin increase under UV-B irradiation, decreasing DNA and protein damage [2]. Two UV-absorbing flavones, maysin and rhamnosylisoorientin, accumulate in maize leaves of high-altitude lines in response to increased UV-B radiation levels. The biosynthesis of these flavones is controlled by a UV-B regulated *P1*-homologous transcription factor expressed in maize leaves [113]. In response to oxidative stress baicalein and its derivatives act as H_2_O_2_ scavengers in *Scutellaria baicalensis* [3]. Hydrogen peroxide is a substrate of peroxidases and a major active compound in the elimination of reactive oxygen species (ROS). In response to an elicitor, baicalein 7-*O*-glucuronide is hydrolyzed to the aglycone baicalein by a β-glucuronidase. 6,7-dehydrobaicalein was then formed through oxidation of the released baicalein by peroxidases. Thus, H_2_O_2_ is effectively consumed and detoxified during the peroxidase reaction [3].

#### 3.1.2. Biotic Protection

Phytoalexins are a heterogeneous group of low molecular mass specialized metabolites with anti-microbial activity [4]. Some phytoalexins flavones include simple aglycones such as luteolin in sorghum resists *Colletotrichum sublineolum* growth [34]), *C*-glycosyl flavones (isoorientin/orientin, and isovitexin/vitexin in cucumber [114] and flax [115] against fungal infection), and *O*-methylated flavone (tricin inhibits the spore germination of fungal pathogens in rice [116,117])

Other than pathogenic microbes, arbuscular mycorrhiza (AM) fungus and nitrogen fixing bacteria *Rhizobium* and *Frankia* provide the most prevalent examples of symbiosis in the plant kingdom [118,119]. Multiple metabolic changes, including the alteration of flavonoid expression in the roots of the host plants, occur before and after the penetration of the host plant’s root by microorganisms [120]. In the Legume family, flavones have been identified as signaling molecules and regulators in the development of root nodulation. In 1986, luteolin and 7,4′-dihydroxyflavone (DHF) released by roots of alfalfa (*Medicago sativa*) and white clover (*Trifolium repens*) were identified as interacting with nodulation (Nod) factor (NodD) to activate transcription of other nodulation genes (*nodABC* and *nodFE*) in *Rhizobium meliloti* and *R. trifolii*, respectively [5,121]. Researchers further demonstrated that DHF in *Medicago truncatula* nodulation are not only important for the induction of *Sinorhizobium meliloti nod* genes in the rhizosphere by released DHF [33], but also plays an essential role for sustaining Nod factor induction during nodulation by root internal DHF [122]. Luteolin-7-*O*-glucoside serves as an attractant of *R. meliloti* resulting in enhanced nodulation, thus indirectly regulating the growth of alfalfa [123]. Flavones also act as regulators in a plant-AM fungus interaction during the pre-colonization and cell-to-cell stages. Chrysin and luteolin showed a stimulatory effect on the pre-symbiotic hyphal grow of several *Glomus* and *Gigaspora* species, and also affected root colonization by increasing the number of entry points of tomato (*Lycopersicum esculentum*) [124]. In melon (*Cucumis melo*) roots, isovitexin 2”-*O*-glucoside accumulated under low phosphate conditions to stimulate mycorrhizal colonization [120]. Apigenin showed a mycorrhiza formation-stimulating activity during root colonization of soybean by the AM fungus *Glomus mosseae* [125].

Plant allelopathy is a biological phenomenon by which plants produce special compounds, including flavones, to interfere with the growth and/or reproduction of other plant species [126]. It is not just plant-plant interference, but can also involve soil-mediated chemical intervention. Tricin was found in rice root exudates to act as an allelochemical inhibiting the growth of the weeds *Echinochloa crus-galli*, *Cyperus difformis* and *C. iris* [127,128,129]. The *C*-glycosyl flavone isoschaftoside from cattle forage legume (*Desmodium uncinatum*) root exudate was identified as an allelochemical capable of inhibiting growth of the parasitic weed *Striga hermonthica* [7,101].

Flavones are also produced by plants as inhibitory compounds against herbivores, including insects and nematodes [1]. We mentioned previously the effects of maize silk maysin on corn earworm and fall armyworm [94,95,96,97]. In insect herbivore (*Spodoptera littoralis* larvae)-damaged alfalfa (*Medicago sativa*) plants, increased levels of apigenin make alfalfa unpalatable to the larvae [130]. Three flavone *C*-glycosides (schaftoside, isoschaftoside, and neoschaftoside) are the main compounds responsible for an ingestion inhibiting activity to the sucking deterrent brown planthopper, *Nilaparvata lugens*, in rice phloem sap [131]. Tricin was identified as anti-feedant to the brown planthopper [132] and boll weevil [133] in rice, and an aphid-feeding deterrent in wheat [134]. Recently, the *C*-glycosyl flavone adonivernith (orientin 2”-*O*-xylopyranoside) was overproduced in the carpel walls of the European globeflower *Trollius europaeus* after infection by *Chiastocheta* fly larvae, resulting in larval growth inhibition [135,136]. Finally, *O*-methyl-apigenin-*C*-deoxyhexoside-*O*-hexoside was found to be induced in oats tissues attacked by the nematodes *Heterodera* and *Pratylenchus* [137].

#### 3.1.3. Plant Development

Copigmentation is a phenomenon that increases color intensity and stability. This process is achieved by the formation of complex associations between pigments (anthocyanins) and other colorless flavonoids (e.g., flavones), or metallic ions [1]. For instance, isovitexin is involved in the bluing effect with anthocyanins on the flower color of Japanese garden iris (*Iris ensata*) [138]; vitexin or orientin enhance the color intensity and the stability of anthocyanins in blueberry juice [139]; and a supramolecular metal complex pigment, metalloanthocyanin, consists of stoichiometric amounts of anthocyanins, flavones, and metal ions with a fix ratio at 6:6:2, respectively [8,140]. Recently, an unusual covalently linked anthocyanin-flavone *C*-glycoside dimer was isolated from the leaves of *Oxalis triangularis* [141]. Unexpectedly, tricin was identified as a monomer in monocot lignins, which are complex phenylpropanoid polymer in plant cell walls [142,143,144,145]. Indeed, during lignification, tricin acts as a nucleation site that initiates lignin polymer chains in monocots [9]. It is yet unclear whether the tricin that participates in lignin formation derives from the general flavonoid pathway, or whether there is a specialized route or flavone pool that feeds into lignin.

### 3.2. Molecular Interactions of Flavones with Other Molecules

In addition to the chemical diversity of flavone structures, different interactions between flavones and other molecules are also critical for fulfilling multiple biological functions in plants. Here, we include some representative interactions, such as those involving lipids, nucleic acids, and proteins [1].

#### 3.2.1. Interactions with Lipids: Flavone-Membrane Interaction

How flavones interact with membrane lipids is mainly determined by the lipophilicity and planar structure of the compounds [146]. This has significant consequences for the ability of flavonoids in general to cross cellular membranes. The relative hydrophobicity of flavones is related to the number and position of -OH groups [147]. By using dipalmitoyl phosphatidyl choline (DPPC) lipid bilayers to represent a biological membrane, the orientation and localization of flavones was demonstrated [148]. The results showed that 5- and 7-hydroxy flavones interact with the lipid surface, while 6-hydroxy flavone and chrysin localize adjacent to the glycerol backbone. It was proposed that flavone -OH groups interact with polar groups on the membrane through H-bonding [147]. By analyzing the interaction of structurally different flavones with membranes, these studies showed that flavone A- or C-rings orient towards the lipid-water interface while the B-ring penetrates into the hydrophobic core [147]. The hydrophilic nature of flavones increases with the number of -OH groups. As flavones become more hydrophilic, their membrane localization shifts towards the aqueous environment [149]. This may explain why apolar flavones, such as chrysin, locate closer to the membrane center, whereas luteolin shows a higher propensity towards the polar region [150]. The studies also showed that the presence of the C-5 -OH enhances flavone lipophilicity [147].

#### 3.2.2. Interactions with Nucleic Acids

The interaction between flavones and DNA is proposed as a mixed mode of intercalation (between the base pairs) and groove binding [151]. Typically, flavones prefer to bind higher order DNA structures such as tri- and tetraplexes [152]. Similar as in the interaction with lipids, -OH substituents were found to be also important for DNA binding. Flavones hydroxylated at the 7 position, such as 7-hydroxyflavone and 5,7-dihydroxyflavone, were identified as the most relevant for DNA binding [153]. Apigenin could intercalate into the base pairs of calf thymus DNA, forming an apigenin-DNA complex largely driven by hydrophobic interactions [154]. The interaction between apigenin and yeast RNA was also investigated. The carbonyl groups of the RNA bases are involved in H-bond formation with apigenin, which also binds RNA through G-C and A-U bases through intercalation [155].

#### 3.2.3. Interactions with Proteins

Flavone-protein interactions have various biological effects. Flavones could bind human serum albumin (HSA) for transportation through plasma [156]. Two flavones, baicalin and baicalein, interacted with HSA in the vicinity of the Trp214 residue which is located in the hydrophobic cavity of subdomain IIA [156]. The binding capacity between flavones and serum albumin is affected by A-ring hydroxylation [157]. As more hydroxyl groups are introduced, the intra-molecular H-bonds within flavones are easily formed, and the affinities for serum albumins decrease [157]. Moreover, chrysin interacts with bovine serum albumin (BSA) through hydrophobic interactions [158]. Flavones also act as modulators of several biological activities. These include inhibitory effects of 8-prenyl-chrysin on the P-glycoprotein activity [159] and of several flavones on CYP-dependent monooxygenases (e.g., chrysin and apigenin on CYP1A2 [160], acacetin and hesperetin on CYP1B1 [161]; and chrysin, apigenin, and acacetin on CYP19 [162]); increasing sensitivity of GABA_A_ receptor by interaction with apigenin [163], chrysin [164,165], or wogonin [164,165]; and competitive inhibition of xanthine oxidase by apigenin and isovitexin [166]. In human breast cancer cells, apigenin can bind to 160 cellular targets, which include the heterogeneous nuclear ribonucleoprotein A2 (hnRNPA2), a factor involved in splicing regulation, mRNA stability, and mRNA transport [167] (see below).

### 3.3. Major Flavone Health Benefits

Flavones are non-essential nutrients that provide additive nutraceutical value to our diet. Their health beneficial activities have been historically recognized across different cultures. Flavonoids, including flavones, have received increasing attention due to their anti-inflammatory, anti-microbial and anti-cancer activities. However, the molecular mechanisms responsible for these activities are just starting to be deciphered.

One of the first beneficial effects ascribed to flavones were anti-oxidant activities, based on the ability of these compounds to scavenge reactive oxygen species (ROS). Structural-functional relationship analyses identified luteolin as one of the most potent inhibitors of xanthine oxidase [168], a key enzyme in ROS production. Reduction of ROS by apigenin prevents endothelial damage during acute inflammation and restores mitochondrial function [169].

Most of the anti-inflammatory and anti-microbial activities attributed to flavones seem to be centered on their ability to regulate the Toll receptor (TLR)/NFκB axis. This is a central pathway in the host-pathogen interplay in mammals [170], responsible for the expression of inflammatory mediators, including tumor necrosis factor α (TNFα), interleukin-1β (IL-1β) and cyclooxygenase-2 (COX-2), an enzyme mediating the conversion of arachidonic acid to prostaglandins. Notably, great similarities are found between the mammalian TLR/NFκB and plant pathogen defense pathways, suggesting that flavones may regulate evolutionary conserved targets [171]. Studies from our group showed that in macrophages and in animal models, apigenin reduces the phosphorylation of the NFκB p65 subunit, required for its transcriptional activity [172,173]. Inhibition of p65 phosphorylation reduces the expression of inflammatory cytokines, limiting the cell damage characteristic of acute inflammation. Other flavones, such as the acacetin and wogonin, abundant in saffron seeds and scutellaria, inhibit COX-2 by halting NFκB nuclear localization [174]. Overall, glycosides show less anti-inflammatory activity than aglycones, probably a consequence of their reduced cellular absorption [175]. Combination of the *C*-glycosyl flavones orientin and isoorientin reduced the production of the inflammatory mediator molecule High Mobility Group Box-1 (HMGB-1), but this effect was not observed when either of the flavones was used alone or in combinations of vitexin and isovitexin, suggesting high specificity in their mechanisms of action [176].

Recent studies identified additional mechanisms responsible of the anti-inflammatory activity of flavones, including the regulation of non-coding RNAs. Large microRNA screenings showed that apigenin, or consumption of celery foods which have a high content of apigenin, reduce microRNA155 (miR155) expression, a main inflammatory regulator [177]. miR155 binds to 3′-UTR regions of several inflammatory cytokines, suggesting an additional mechanism by which flavones can restore homeostasis during acute inflammation, independent of their anti-oxidant activity. So far, studies in Arabidopsis indicate that the accumulation of anthocyanin follows patterns regulated by miR156 [178], but whether flavones or other flavonoids themselves induce miRs in plants is yet to be determined.

Consistent with the ability of flavones to regulate inflammation, interventions with the Mediterranean diet, which is rich in flavonoids, showed improved cardiac function, reduced hypertension and obesity [179,180]. Epidemiological studies highlighted the beneficial effects of this diet in metabolic function [181].

Flavones also affect leukocyte migration. This has profound effects in both inflammation and cancer. Luteolin reduces Rho GTPases activity, decreasing leukocyte migration thereby resulting in the prevention of inflammation and neuronal damage [182]. Apigenin inhibits leukocyte migration by affecting the Janus kinase 3 (JAK3), a non-receptor tyrosine kinase [183]. Flavones’ ability to reduce cell migration has great impact in cancer, suggesting alternative therapeutic approaches to reduce metastasis. Apigenin reduced breast cancer cell migration, by inhibiting mitogen activated protein kinases (MAPK), including ERK and JNK [184].

Flavones’ anti-carcinogenic activity promotes apoptosis of cancer cells at doses that are cell-type specific. Leukemias in general seem more susceptible to flavones, undergoing caspase-dependent apoptosis at low micromolar ranges [185]. In contrast, higher flavone concentrations are needed to induce apoptosis of cancer cell lines from solid tumors including prostate, lung and skin cancer [185,186,187]. The anti-carcinogenic effect of flavones is given in part by their ability to induce DNA damage, and is accompanied by cell cycle arrest at G1 or G2, depending on the particular cell type. Interestingly, the ability of apigenin to induce cell death in cancer cells is independent of ROS production [188], supporting a beneficial role of flavones independent of their anti-oxidant activity. Flavones, such as apigenin, induce the phosphorylation of heat shock protein 27, an inhibitor of apoptosis highly expressed in cancers, by promoting the activity of the p38 kinase [189]. Yet, this effect is not direct and the direct target remains to be identified. Maysin induces apoptosis of PC-3 prostate cancer cells, by the mitochondrial-intrinsic pathway, but had no effect in lung, colon or stomach cancer cell lines [190]. Anti-cancer activity may also be due to inhibition of the NFκB pathway. *In silico* predictions suggest that apigenin associates with IKKα, a kinase upstream of NFκB [191]. However, in models of acute inflammation, apigenin reduces IKKβ without affecting IKKα [172]. Additional experiments will be needed to further understand the specific contributions of the molecular networks responsible for the anti-carcinogenic effects of apigenin and other flavones. Higher levels of NFκB activity and COX-2 are common in both cancers and acute inflammation, suggesting shared mechanisms of action. Identification of the direct targets will highly contribute to understand the molecular mechanism related to flavones and health. The use of PD-Seq (phage display high-throughput sequencing), a novel approach for small target identification, identified several targets, suggesting that dietary compounds, unlike pharmaceuticals, may target several molecules. Many of the identified targets were validated using independent strategies, suggesting that most are biologically relevant targets [167]. Using PD-Seq, we found that apigenin associates with several RNA binding proteins, including hnRNPA2. hnRNPA2 regulates alternative splicing and is highly expressed in tumors. The treatment of breast cancer cells with apigenin changed aberrant splicing isoforms found in cancer cells to isoforms commonly found in non-malignant cells. These results highlight the existence of additional mechanisms involved in the health beneficial effects of flavones and prompt the need of future studies in the area.

## 4. Conclusions and Future Prospects

From all of the above, it is clear that flavones are not only important compounds for the biology of plants and for human health, but they also provide convenient specialized metabolites to better understand the chemical complexity of plants, and how different enzymes have evolved to use the same substrates to produce identical products, sometimes in the same organism. The characterization of the flavone pathway in a number of different plant species provides a valuable resource of clones and enzymes for metabolic engineering to produce organisms with enhanced tolerance to biotic and abiotic stress conditions, or for improved animal and human nutrition. Finally, the identification of proteins that specifically interact with flavones provides the next frontier in establishing how related specialized metabolites can have such a diversity of biological activities.

## Figures and Tables

**Table 1:** Overview of functionally characterized plant FNSI, FNSII, and F2H enzymes.

Protein	Accession No.	Species	Function	Ref.
CYP93B1	AB001380	*Glycyrrhiza echinata* (Licorice)	F2H	[32]
CYP93B2	AF156976	*Gerbera hybrida*	FNSII	[37]
CYP93B3	AB028151	*Antirrhinum majus* (Snapdragon)	FNSII	[28]
CYP93B4	AB028152	*Torenia hybrida*	FNSII	[38]
CYP93B6	AB045592	*Perilla frutescens var. Crispa*	FNSII	[39]
CYP93B10	DQ354373	*Medicago truncatula* (Barrelclover)	F2H	[33]
CYP93B11	DQ335809	*Medicago truncatula* (Barrelclover)	F2H	[33]
CYP93B16	GU658027	*Glycine max* (Soybean)	FNSII	[29]
CYP93B24	KT963453	*Scutellaria baicalensis*	FNSII	[40]
CYP93B25	KT963453	*Scutellaria baicalensis*	FNSII	[40]
CYP93G1	AK100972	*Oryza sativa* (Rice)	FNSII	[30]
CYP93G2	AK099468	*Oryza sativa* (Rice)	F2H	[35]
CYP93G3	XP_002461286	*Sorghum bicolor* (Sorghum)	F2H	[34]
CYP93G5	GRMZM2G167336	*Zea mays* (Maize)	F2H	[36]
LjFNSII-1.1	KU127576	*Lonicera japonica*	FNSII	[31]
LjFNSII-2.1	KU127578	*Lonicera japonica*	FNSII	[31]
LmFNSII-1.1	KU127580	*Lonicera macranthoides*	FNSII	[31]
PcFNSI	AY230247	*Petroselinum crispum* (Parsley)	FNSI	[19]
DcFNSI	AY817675	*Daucus carota* (Wild carrot)	FNSI	[21]
AgFNSI	AY817676	*Apium graveolens* (Celery)	FNSI	[21]
CmFNSI	AY817677	*Conium maculatum*	FNSI	[21]
AcFNSI	DQ683350	*Aethusa cynapium*	FNSI	[20]
AaFNSI	DQ683352	*Angelica archangelica* (Wild celery)	FNSI	[16]
CcFNSI	DQ683349	*Cuminum cyminum*	FNSI	[16]
OsFNSI-1	NP_922524	*Oryza sativa* (Rice)	FNSI	[22]
PaFNSI	KJ439220	*Plagiochasma appendiculatum* (Liverwort)	FNSI/F2H	[23]
ZmFNSI-1	GRMZM2G099467	*Zea mays* (Maize)	FNSI	[24]
AtDMR6	AT5G24530	*Arabidopsis thaliana* (Arabidopsis)	FNSI	[24]

## Abbreviations

The following abbreviations are used in this review:

PAL	phenylalanine ammonia-lyase
C4H	cinnamic acid 4-hydroxylase
CoA	coenzyme A
4CL	*p*-coumaroyl: CoA ligase
4CL-like	cinnamic acid specific CoA ligase
CHS	chalcone synthase
CHI	chalcone isomerase
FNS	flavone synthase
2-ODD	Fe^2+^/2-oxoglutarate-dependent dioxygenase
F2H	flavanone-2-hydroxylase
F3′H	flavanone-3′-hydroxylase
F6H	flavanone-6-hydroxylase
CYP	cytochrome P450
IFS	isoflavone synthase
UGTs	UDP-glycosyltransferases
CGT	*C*-glycosyl transferase
OGT	*O*-glycosyl transferase
HID	2-hydroxyisoflavanone dehydratase
OMT	*O*-methyltransferase
FOMT	flavonoid *O*-methyltransferase
RHM	UDP-rhamnose synthase
